# Vascular protection afforded by zinc supplementation in human coronary artery smooth muscle cells mediated by NRF2 signaling under hypoxia/reoxygenation

**DOI:** 10.1016/j.redox.2023.102777

**Published:** 2023-06-07

**Authors:** Fan Yang, Matthew J. Smith, Alexander Griffiths, Alexander Morrell, Sarah J. Chapple, Richard C.M. Siow, Theodora Stewart, Wolfgang Maret, Giovanni E. Mann

**Affiliations:** a King's British Heart Foundation Centre of Research Excellence, School of Cardiovascular and Metabolic Medicine & Sciences, Faculty of Life Sciences & Medicine, King's College London, 150 Stamford Street, London, SE1 9NH, UK; b London Metallomics Facility, Faculty of Life Sciences & Medicine, King's College London, UK; c Research Management & Innovation Directorate (RMID), King’s College London, UK; d Departments of Biochemistry and Nutritional Sciences, School of Life Course & Population Sciences, Faculty of Life Sciences & Medicine, King's College London, UK

**Keywords:** Coronary artery smooth muscle cells, Metals, Metallomics, Metallothionein, Zinc, NRF2, Redox status, Physiological normoxia, Hyperoxia, Hypoxia, Oxygen, Hypoxia-reoxygenation

## Abstract

Zinc (Zn) has antioxidant, anti-inflammatory and anti-proliferative actions, with Zn dysregulation associated with coronary ischemia/reperfusion injury and smooth muscle cell dysfunction. As the majority of studies concerning Zn have been conducted under non-physiological hyperoxic conditions, we compare the effects of Zn chelation or supplementation on total intracellular Zn content, antioxidant NRF2 targeted gene transcription and hypoxia/reoxygenation-induced reactive oxygen species generation in human coronary artery smooth muscle cells (HCASMC) pre-adapted to hyperoxia (18 kPa O_2_) or normoxia (5 kPa O_2_). Expression of the smooth muscle marker SM22-α was unaffected by lowering pericellular O_2_, whereas calponin-1 was significantly upregulated in cells under 5 kPa O_2_, indicating a more physiological contractile phenotype under 5 kPa O_2_. Inductively coupled plasma mass spectrometry established that Zn supplementation (10 μM ZnCl_2_ + 0.5 μM pyrithione) significantly increased total Zn content in HCASMC under 18 but not 5 kPa O_2_. Zn supplementation increased metallothionein mRNA expression and NRF2 nuclear accumulation in cells under 18 or 5 kPa O_2_. Notably, NRF2 regulated HO-1 and NQO1 mRNA expression in response to Zn supplementation was only upregulated in cells under 18 but not 5 kPa. Furthermore, whilst hypoxia increased intracellular glutathione (GSH) in cells pre-adapted to 18 but not 5 kPa O_2_, reoxygenation had negligible effects on GSH or total Zn content. Reoxygenation-induced superoxide generation in cells under 18 kPa O_2_ was abrogated by PEG-superoxide dismutase but not by PEG-catalase, and Zn supplementation, but not Zn chelation, attenuated reoxygenation-induced superoxide generation in cells under 18 but not 5kPaO_2_, consistent with a lower redox stress under physiological normoxia. Our findings highlight that culture of HCASMC under physiological normoxia recapitulates an *in vivo* contractile phenotype and that effects of Zn on NRF2 signaling are altered by oxygen tension.

## Highlights

• Physiological normoxia upregulates calponin-1 expression in coronary artery smooth muscle cells.
• Physiological normoxia attenuates zinc accumulation following Zn supplementation.
• Zinc supplementation activates NRF2 signaling and HO-1, NQO1 and MT-1 mRNA expression.
• Zinc supplementation attenuates hypoxia/reoxygenation induced superoxide generation.

## Graphical abstract

**Figure:**
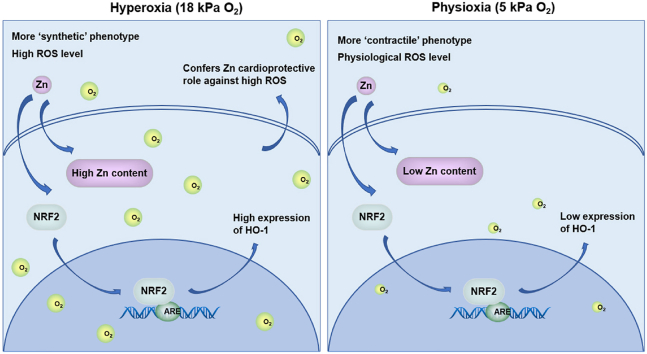

## 1 Introduction

Zinc (Zn) has emerged as a valuable biomarker in diagnosis and therapy in coronary artery and heart disease associated with oxidative stress and redox dysregulation [[1], [2], [3]]. Nuclear factor E2-related factor 2 (NRF2) is a well-known antioxidant transcription factor [[4], [5], [6], [7], [8]] and plays a critical role in the maintenance of cellular redox homeostasis in oxidative stress and ischemia-reperfusion injury [9,10]. Although Zn supplementation has been reported to reduce superoxide (O_2_^•-^) generation, lower apoptotic indices, restore ATP levels and attenuate NADPH oxidase mediated oxidative stress [2,[11], [12], [13], [14]], there is evidence that Zn supplementation at the onset of reperfusion enhances the severity of myocardial infarction (MI) [15] and increases reactive oxygen species (ROS) generation in cardiomyocytes and aortic smooth muscle cells [[16], [17], [18]]. We recently established that NRF2 activation regulates total Zn content in coronary vascular cells in a cell-type specific manner under physiological oxygen levels [19]. Based on these findings and conflicting reports on Zn supplementation, further investigation of the relationship between Zn and NRF2 targeted cellular antioxidant defenses in IR and hypoxia/reoxygenation is warranted.

As a redox-inactive metal, Zn plays an important role in oxidative stress by affording some protection of thiol groups against reactive oxygen species (ROS) damage [20,21]. NRF2 transcriptional activity is increased by ROS interacting with a zinc coordination site in Keap1 [22], leading to inhibition of NRF2 ubiquitination and proteasomal degradation [23,24]. In human renal tubular cells, Zn supplementation induced inhibition of GSK3β has been linked to an inhibition of NRF2 nuclear export and enhanced transcriptional activity [25]. Zn has also been shown to regulate expression of the NRF2 target glutamate-cysteine ligase, the rate-limiting enzyme in glutathione synthesis [26]. Moreover, Zn can activate the metal regulatory element binding transcription factor 1 (MTF-1), which plays an important role in regulating antioxidant responses and maintaining metal homeostasis [27]. Activation of MTF-1 upregulates expression of metallothioneins (MT) and the selenoprotein1 gene, which encodes an antioxidant glutathione-binding protein known to scavenge free radicals [27]. MTF-1 and NRF2 transcription factors are thus linked through a pool of free Zn^2+^ modulated by both MT and transcriptional machinery [28].

We recently highlighted cell-type differences in redox signaling and total intracellular Zn content in human coronary artery endothelial and smooth muscle cells (HCASMC) upon lowering pericellular O_2_ levels from standard cell culture hyperoxia (18 kPa) to physiological normoxia (5 kPa) and hypoxia (1 kPa) [19]. In the present study, we investigate whether Zn chelation or supplementation affect intracellular Zn content and NRF2 redox signaling differentially in HCASMC cultured long-term (5 days) under hyperoxia or physiological normoxia in an O_2_-controlled workstation. In the context of hypoxia/reoxygenation, we report the first evidence that Zn supplementation significantly attenuates reoxygenation induced ROS generation in HCASMC under 18 but not 5 kPa O_2_. In support of our recent recommendations [29,30], the present study further highlights the importance of assessing redox signaling, Zn metabolism and the effects of Zn supplementation in cells cultured under pericellular O_2_ levels encountered *in vivo*.

## 2 Methods and materials

### 2.1 HCASMC culture under defined pericellular O_2_ levels

As previously described [19], primary human coronary artery smooth muscle cells (HCASMC, PromoCell, Germany) were cultured in Smooth Muscle Cell Basal Medium 2 (PromoCell), supplemented with growth medium 2 supplement pack (PromoCell) and 1% penicillin (100U/ml)/streptomycin (100 μg/ml). Cells were treated with medium containing 2 μM Zn or with medium supplemented with a Zn chelator (TPEN, 1.25 μM) or ZnCl_2_ (10 μM) + pyrithione (Zn ionophore, 0.5 μM). HCASMC were pre-adapted for 5d in a dual Scitive O_2_-controlled workstation (Baker, USA) under 18 kPa O_2_ (hyperoxia) or 5 kPa O_2_ (physiological normoxia) and 5% CO_2_ at 37 °C. All protocols and experiments were conducted within an O_2_-controlled workstation and/or plate reader (CLARIOstar, BMG Labtech, Germany) [[31], [32], [33], [34]].

### 2.2 Measurement of proliferation using real time cell analysis (RTCA) platform, cell number and cell protein

Cell proliferation was assessed using an RTCA (iCELLigence™, Acea Biosciences) platform [31], which uses non-invasive electrical impedance to quantify cell proliferation label-free in real-time. HCASMC adapted to 18 or 5 kPa O_2_ were seeded into E-Plates® in triplicate at a concentration of 7000 cells/well. Adherent cells at the electrode-solution interface impede electron flow, and the magnitude of impedance (Cell Index, CI) serves as an index of cell proliferation. Cell adhesion was measured over the first 2 h to achieve a baseline CI and proliferation then recorded over 6 days with media changed every 2d. As previously described [34], proliferation was also assessed by measuring cell number and total protein content during 1 – 5 days culture under 18 or 5 kPa O_2_.

### 2.3 Inductively coupled plasma mass spectrometry analysis of total intracellular Zn content in HCASMC

We previously described ICP-MS protocols for measuring total intracellular Zn content in HCASMC lysates collected in purified trace metal free water with a resistivity ≥18.2 MΩcm obtained from a Milli-Q system (Merck Millipore, USA) [19]. Cell lysates were introduced to an ICP-QMS via a Cetac ASX-520 autosampler (Teledyne, USA) coupled to a SeaSpray glass nebulizer fitted to a quartz cyclonic spray chamber. Zn concentrations were normalized to cell protein measured using the bicinchoninic acid (BCA) assay.

### 2.4 NRF2 nuclear accumulation assessed by immunofluorescence

To assess nuclear translocation of NRF2 after treatment with a Zn chelator or Zn supplementation, HCASMC were pre-adapted for 5d to 18 or 5 kPa O_2_ and then seeded into 8-chambered coverslips (Ibidi, Germany) for 48 h. Cells were then treated for 16 h with (i) vehicle (Veh, 0.01% dimethyl sulfoxide (DMSO, Sigma-Aldrich, UK), (ii) N,N,N′,N'-tetrakis (2-pyridylmethyl) ethylenediamine (Zn chelator, TPEN, 1.25 μM, Sigma-Aldrich, UK) [35] or (iii) ZnCl_2_ (10 μM, Sigma-Aldrich, UK) and 2-mercaptopyridine N-oxide sodium salt (Zn^2+^ ionophore pyrithione, 0.5 μM, Sigma-Aldrich, UK). Immunofluorescence analysis was performed using a primary anti-NRF2 antibody (Santa Cruz, USA) and donkey anti-Rabbit DyLight® 488 conjugated secondary antibody (Bethyl Laboratories, USA). Fluorescence was visualized at × 40 magnification using a fluorescence microscope (Etaluma LS720, USA), with images quantified as the ratio of nuclear:cytoplasmic NRF2 immunofluorescence and cell nuclei stained with DAPI [36].

### 2.5 Immunoblotting

Whole cell lysates were collected using SDS lysis buffer supplemented with protease inhibitor cocktail (Sigma-Aldrich, UK), separated by gel electrophoresis, electro-transferred onto polyvinylidene difluoride membranes (Millipore, Sigma, USA) and probed with primary and HRP-conjugated secondary antibodies (Millipore, Sigma, USA): NQO1 (Santa Cruz, USA), HO-1 (BD Biosciences, USA), ZnT1 (Abcam, UK) and β-actin (Sigma-Aldrich, UK) and analyzed by enhanced chemiluminescence (Millipore, Sigma, USA). Images were captured using a G:Box system (Syngene, UK) and densitometric analysis conducted using ImageJ software (National Institutes of Health, USA), as previously described [19].

### 2.6 Quantitative RT-PCR

HCASMC RNA was isolated using a RNeasy® Mini Kit (Qiagen, Germany) and RNA content and purity assessed using a spectrophotometer (NanoDrop Technologies, USA) [31]. Total RNA was reverse–transcribed using a High Capacity cDNA Reverse Transcription Kit (Applied Biosystems, USA). HO-1, NQO1 and MT-1 mRNA were assessed by real-time qPCR (Applied Biosystems, USA) and normalized to the geometric mean of ribosomal protein lateral stalk subunit P0 (RPLP0), TATA-binding protein (TBP) and succinate dehydrogenase complex, subunit A (SDHA) (primer sequences in Supplementary Table 1).

### 2.7 L-012 chemiluminescence measurements of ROS generation in HCASMC under hypoxia/reoxygenation

HCASMC were seeded into white clear bottomed 96-well plates in quadruplicate and adapted for 5d under 18 or 5 kPa O_2_. As previously described [34], cell monolayers were incubated in the absence or presence of polyethylene glycol superoxide dismutase (pSOD, 20U/ml, Sigma-Aldrich, UK) or polyethylene glycol catalase (pCAT, 200U/ml, Sigma-Aldrich, UK) to scavenge superoxide or H_2_O_2_ respectively. To determine the effects of Zn chelation or supplementation on ROS generation, cells were pre-adapted for 5d to 18 or 5 kPa O_2_ and then treated for 16 h with (i) vehicle (Veh, 0.01% DMSO), (ii) TPEN (1.25 μM) or (iii) ZnCl_2_ (10 μM ZnCl_2_ + pyrithione 0.5 μM). After incubation with the chemiluminescent luminol analogue L-012 (8-amino-5-chloro-7-phenylpyridol[3,4-d] pyridazine-1,4-(2H,3H)dione sodium salt, 10 μM, Tocris Bioscience, UK), cells were rapidly transferred from the O_2_-controlled workstation to an O_2_-controlled plate reader (CLARIOstar, BMG Labtech, UK) at 37 °C [34]. Cells were then exposed to hypoxia (1 kPa O_2_) for 1 h and reoxygenation under either 18 or 5 kPa O_2_, respectively, and chemiluminescence measured at 60s intervals over 3 h and expressed as mean light units × 10^6^ or 10^4^/mg protein.

L-012 is widely used to measure superoxide (O_2_^.−^) and other reactive oxygen species (ROS) in biological systems [37]. Although O_2_^.−^ alone does not react with L-012 to emit luminescence, oxidation of the probe to its radical and reaction of the luminol radical with self-generated O_2_^.−^ during oxidation of L-012 leads to the emission of blue light, which can be inhibited by superoxide dismutase (SOD) [37].

### 2.8 Effects of hypoxia/reoxygenation on intracellular glutathione levels

HCASMC were pre-adapted for 5d to 18 or 5 kPa O_2_ in an O_2_-regulated workstation and then exposed to hypoxia for 1 h by lowering O_2_ in the workstation to 1 kPa and reoxygenation for 1 h or 24 h under 18 or 5 kPa O_2_, respectively (see Supplementary Fig. 3). GSH levels were determined using a fluorometric assay [38]. Luminescence and fluorescence were measured in a plate reader (CLARIOstar, BMG Labtech, Germany) and expressed as nmol/mg protein, as previously described [31].

### 2.9 Effects of ZnCl_2_ and pyrithione co-treatment on cell viability

Cell viability was assessed using 3-(4,5-dimethyl-thiazol-2-yl)-2,5-diphenyltetrazolium bromide (MTT, Sigma-Aldrich, U.K.), as previously described [39]. HCASMC were co-treated for 16 h with ZnCl_2_ (10, 12 or 14 μM) and the Zn ionophore pyrithione (0.25, 0.5, 0.75 or 1 μM) and then incubated with 5 μg/ml MTT for 3 h at 37 °C. Insoluble formazan salts were dissolved in DMSO and absorbance at 570 nm measured in a plate reader (CLARIOstar, BMG Labtech, Germany).

### 2.10 Statistical analysis

Data denote the mean ± S.E.M. of 3–6 independent HCASMC cultures and were analyzed using Graphpad Prism 8. Significance was assessed using either an unpaired Student's *t*-test or one- or two-way ANOVA followed by a Bonferroni Post Hoc test where appropriate, with *P < 0.05, **P < 0.01, ***P < 0.001 and ****P < 0.0001 considered significant.

## 3 Results

### 3.1 Adaptation to defined pericellular O_2_ levels alters HCASMC phenotype and proliferation

**Fig. 1:**
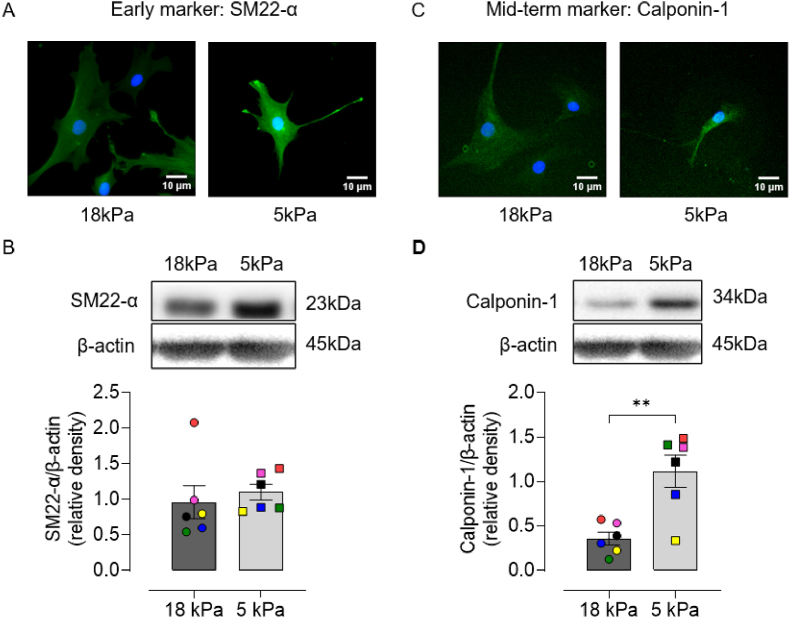
Long-term culture of HCASMC under physiological normoxia (5 kPa O_2_) upregulates calponin-1 expression **A and C,** Representative immunofluorescence images of SM22-α or calponin-1 and DAPI stained nuclei in HCASMC pre-adapted for 5d to 18 or 5 kPa O_2_. **B and D,** Representative immunoblots and densitometric analysis of SM22-α and calponin-1 expression relative to β-actin. Data denote mean ± S.E.M., n = 3–6 independent cell cultures (color-coded), unpaired Student's *t*-test, **P < 0.01. (For interpretation of the references to color in this figure legend, the reader is referred to the Web version of this article.)

Vascular smooth muscle exist in a contractile or a synthetic phenotype, and the degree of differentiation can be detected by the expression of specific markers [40]. Markers of contractile phenotype are divided into early (SMαA, myocardin and SM22-α), mid-term (H-caldesmon and calponin-1) and late (SMMHC-1 and -2 and smoothelin) based on their appearance during embryonic development or differentiation of stem cells toward vascular smooth muscle cells [[41], [42], [43]]. To characterize the effect of pericellular O_2_ on the phenotype of HCASMC, contractile markers were examined by immunostaining and immunoblotting. Representative fluorescence images of SM22-α and calponin-1 staining of HCASMC adapted to 18 or 5 kPa O_2_ are shown in Fig. 1A and C. Although protein expression of SM22-α was affected negligibly under both O_2_ levels (Fig. 1B), calponin-1 expression was significantly increased in HCASMC under 5 kPa O_2_ (Fig. 1D), suggesting that cells cultured long-term under physiological normoxia (5 kPa O_2_), in the absence of HIF-α stabilization [19], exhibit a more contractile phenotype.

**Fig. 2:**
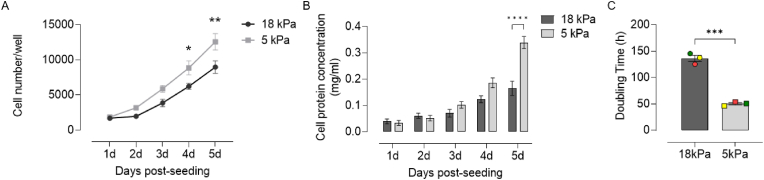
Proliferation of HCASMC during long-term culture under 18 or 5 kPa O_2_ HCASMC were pre-adapted for 5d to either 18 or 5 kPa O_2_. **A-B,** Cells seeded at 3500 cells/well into 96-well plates and cell number counted and total protein content measured over 1–5 days in culture. **C**, Cells seeded at 7000 cells/well in E-Plates® and doubling time monitored continuously over 6 days using an iCELLigence platform. Data denote mean ± S.E.M., n = 3–4 independent cultures (color-coded), two-way ANOVA followed by a Bonferroni Post Hoc test analysis, *P < 0.05, **P < 0.01, ***P < 0.001. (For interpretation of the references to color in this figure legend, the reader is referred to the Web version of this article.)

To further characterize HCASMC during long-term culture under 18 or 5 kPa O_2_, we monitored cell proliferation by determining cell number, total cell protein and changes in bioimpedance using a RTCA platform (Fig. 2). Proliferation of HCASMC was enhanced under 5 kPa compared to 18 kPa O_2_, as evidenced by increased cell number and protein after 5d in culture (Fig. 2A-B) and a significantly reduced doubling time (Fig. 2C).

### 3.2 Zn supplementation increases Zn content in HCASMC under 18 but not 5 kPa O_2_

**Fig. 3:**
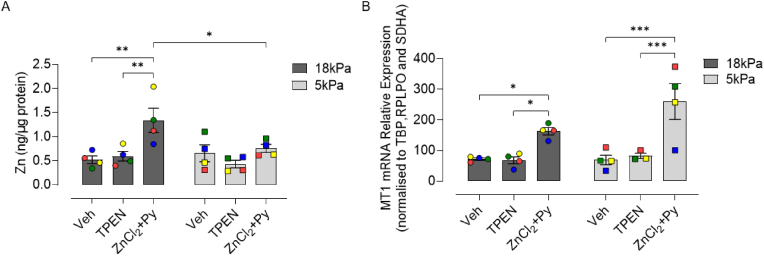
Total intracellular Zn content and metallothionein mRNA expression in HCASMC treated with TPEN or supplemented with Zn under 18 or 5 kPa O_2_ HCASMC were pre-adapted for 5d to 18 or 5 kPa O_2_ and then treated with vehicle (Veh, 0.01% DMSO), TPEN (Zn^2+^ chelator, 1.25 μM) or ZnCl_2_ (10 μM) and 2-mercaptopyridine N-oxide sodium salt (Zn ionophore pyrithione, 0.5 μM) (ZnCl_2_+Py). **A**, ICP-MS analysis of total Zn content in HCASMC following treatment for 16 h with Veh, TPEN or ZnCl_2_+Py. Data denote mean ± S.E.M., n = 4 independent cultures (color-coded), two-way ANOVA followed by Bonferroni's multiple comparisons test, *P < 0.05, **P < 0.01, ***P < 0.001. (For interpretation of the references to color in this figure legend, the reader is referred to the Web version of this article.)

ICP-MS analysis was employed to determine whether total intracellular Zn content in HCASMC pre-adapted for 5d to 18 or 5 kPa O_2_ is affected differently by Zn chelation or supplementation. Basal Zn content was affected negligibly in HCASMC under the two O_2_ levels (18 kPa = 0.52 ± 0.08 ng/μg protein *versus* 5 kPa = 0.66 ± 0.17 ng/μg protein), confirming our previous findings in coronary smooth muscle cells [19]. Treatment of cells with the Zn chelator TPEN (1.25 μM) had no effect on cell viability (Supplementary Fig. 1) and moreover negligible effects on Zn content under either 18 or 5 kPa O_2_ (Fig. 3A). Pyrithione, an ionophore for Zn [44], facilitates cellular uptake of Zn in cardiomyocytes and other cell types [44,45]. We initially assessed the viability of HCASMC following co-treatment with different ZnCl_2_ (0–20 μM) and pyrithione (0–1 μM) concentrations and selected concentrations of ZnCl_2_ (10 μM) + pyrithione (0.5 μM) for all subsequent experiments (Supplementary Fig. 1). As shown in Fig. 3A, Zn supplementation significantly increased Zn content in cells adapted to 18 kPa O_2_ (Veh: 0.52 ± 0.08 ng/μg protein *versus* ZnCl_2_+Py: 1.34 ± 0.26 ng/μg protein) but not in cells adapted to 5 kPa O_2_ (0.66 ± 0.17 ng/μg protein *versus* 0.76 ± 0.08 ng/μg protein). Under the same experimental conditions, Zn supplementation significantly increased metallothionein (MT-1) mRNA in cells under 18 or 5 kPa O_2_ (Fig. 3B).

### 3.3 Effects of Zn supplementation on NRF2 signaling in HCASMC under 18 or 5 kPa O_2_

**Fig. 4:**
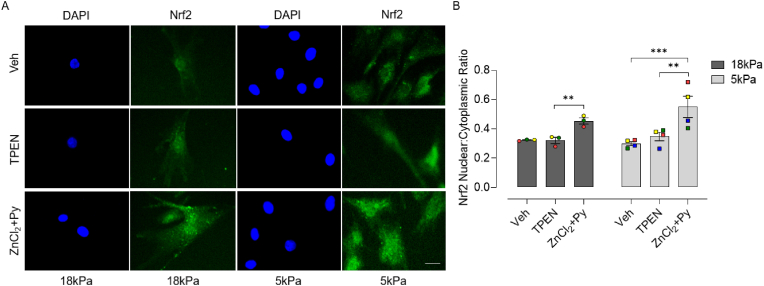
Zn supplementation induces NRF2 nuclear accumulation in HCASMC under 18 or 5 kPa O_2_ A, Representative NRF2 positive immunofluorescence and DAPI stained nuclei in HCASMC pre-adapted to18 or 5 kPa O_2_ and then treated for 16 h with Veh (0.01% DMSO), TPEN (1.25 μM) or ZnCl_2_ (10 μM) + pyrithione (Py, 0.5 μM), respectively. B, Quantification of NRF2 nuclear:cytoplasmic ratio in HCASMC treated with Veh, TPEN or ZnCl_2_+Py. Data denote mean ± S.E.M., n = 3–4 independent cultures (color-coded) with 20–30 cells analyzed in each culture, two-way ANOVA followed by a Bonferroni Post Hoc test analysis, **P < 0.01, ***P < 0.001. Scale bar = 20 μm. (For interpretation of the references to color in this figure legend, the reader is referred to the Web version of this article.)

**Fig. 5:**
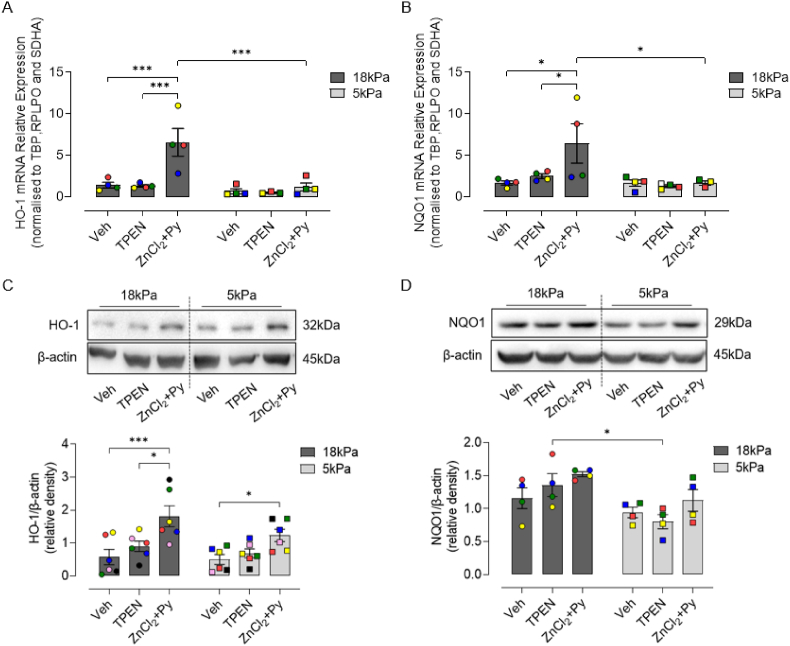
Effect of Zn supplementation on HO-1 and NQO1 mRNA and protein expression in HCASMC under 18 or 5 kPa O_2_ HCASMC were pre-adapted for 5d to 18 or 5 kPa O_2_. **A and B**, mRNA expression of HO-1 and NQO1 in HCASMC treated for 6 h with Veh (0.01% DMSO), TPEN (1.25 μM) or ZnCl_2_ (10 μM) + pyrithione (Py, 0.5 μM), respectively. Values normalized to three housekeeping genes (TBP, RPLPO and SDHA). **C and D**, Representative immunoblots and densitometric analysis of HO-1 and NQO1 expression relative to β-actin following treatment for 16 h with Veh (0.01% DMSO), TPEN (1.25 μM) or ZnCl_2_ (10 μM) + pyrithione (Py, 0.5 μM). Data denote mean ± S.E.M., n = 4–6 independent cultures (color-coded), two-way ANOVA followed by Bonferroni's multiple comparisons test, *P < 0.05, ***P < 0.001. (For interpretation of the references to color in this figure legend, the reader is referred to the Web version of this article.)

We next examined whether Zn chelation or supplementation influences NRF2 nuclear accumulation and HO-1 and NQO1 mRNA/protein expression in cells pre-adapted for 5d to 18 or 5 kPa O_2_. Although chelation of Zn had negligible effects on NRF2 nuclear:cytoplasmic ratios, Zn supplementation significantly enhanced NRF2 nuclear accumulation in cells under 18 and 5 kPa O_2_ (Fig. 4). As shown in Fig. 5, TPEN had negligible effects on HO-1 and NQO1 mRNA/protein expression in cells cultured in complete medium containing low Zn (2.0 μM). In contrast, Zn supplementation significantly increased HO-1 and NQO1 mRNA expression in HCASMC pre-adapted to 18 kPa O_2_, which was markedly attenuated in cells under 5 kPa O_2_ (Fig. 5 A and B). Furthermore, HO-1 protein expression in response to Zn supplementation was attenuated in HCASMC under 5 kPa O_2_ (Fig. 5C). Taken together these data suggest that NRF2 signaling is activated by Zn supplementation in cells adapted to 18 kPa O_2_ and to a lesser extent under 5 kPa O_2_, independent of NRF2 nuclear accumulation.

### 3.4 Effects of hypoxia/reoxygenation on ROS generation, intracellular Zn and glutathione

**Fig. 6:**
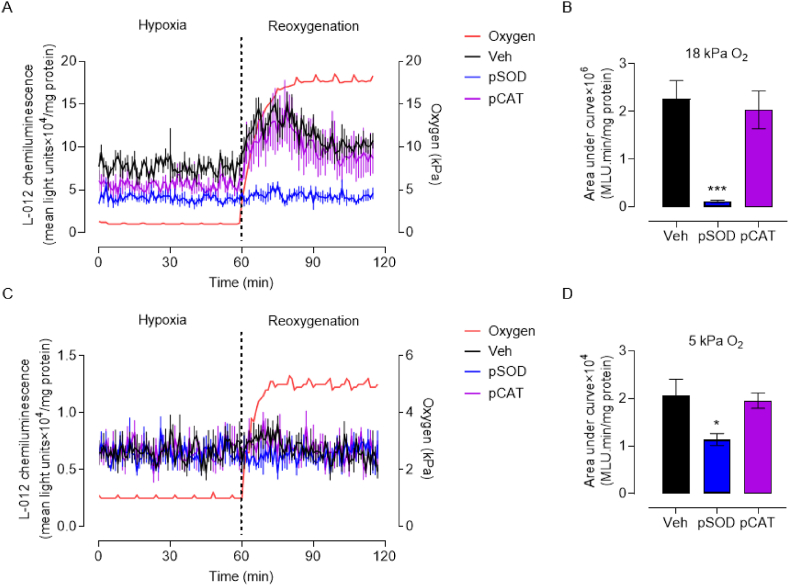
Reoxygenation induced reactive oxygen species generation in HCASMC under 18 or 5 kPa O_2_ **A and C**, Representative L-012 luminescence traces in HCASMC pre-adapted for 5d to 18 or 5 kPa O_2_. Cells were treated with Veh (0.01% DMSO), PEG-superoxide dismutase (pSOD, 20U/ml) or PEG-catalase (pCAT, 200U/ml). Cells were then incubated with L-012 and transferred to an O_2_-regulated plate reader gassed with 18 or 5 kPa O_2_, respectively. L-012 luminescence was detected in the plate reader, with O_2_ reduced to 1 kPa and reoxygenation under 18 or 5 kPa O_2_, respectively. The red line indicates the pericellular O_2_ levels within the plate reader. **B and D**, Area under curve summary of reoxygenation-induced L-012 luminescence changes. L-012 signal in panel B (18 kPa O_2_) denotes area under the curve x10^6^ and in panel D (5 kPa O_2_) area under the curve x10^4^. Data denote mean ± S.E.M., n = 6 independent cultures, one-way ANOVA followed by Bonferroni's multiple comparisons test, *P < 0.05, ***P < 0.001. (For interpretation of the references to color in this figure legend, the reader is referred to the Web version of this article.)

Ischemia and hypoxia induced damage is exacerbated during reperfusion/reoxygenation and has been attributed to an increased generation of ROS [46]. To mimic reactive oxygen species (ROS) generation during reoxygenation, HCASMC were pre-adapted for 5d to 18 or 5 kPa O_2_, loaded with the luminescence probe L-012 and exposed to hypoxia (1 h) and reoxygenation. As shown in Fig. 6A and B, ROS generation during reoxygenation of HCASMC under 18 kPa O_2_ was abrogated by PEG-superoxide dismutase, whilst PEG-catalase had a negligible effect, implicating superoxide anions in the reoxygenation-induced free radical burst. In contrast, reoxygenation-induced free radical generation was negligible in cells adapted to 5 kPa O_2_ (Fig. 6C and D), consistent with our previous findings in brain microvascular endothelial cells [34].

We next determined whether intracellular Zn content and glutathione (GSH) levels were affected differently by hypoxia/reoxygenation in HCASMC pre-adapted to 18 or 5 kPa O_2_. Total intracellular Zn content was not altered by hypoxia (1 h) nor in response to reoxygenation of cells for 1 or 9 h under either 18 or 5 kPa O_2_, respectively (Supplementary Fig. 2). Hypoxia increased intracellular GSH levels in HCASMC pre-adapted to 18 kPa but not 5 kPa O_2_, with levels returning to basal values over 24 h reoxygenation (Supplementary Fig. 3). Our findings contrast with negligible changes in GSH in mouse peritoneal macrophages cultured under 18 kPa O_2_ and exposed to hypoxia for 1 h [47].

### 3.5 Effects of hypoxia/reoxygenation on NQO1, GCLM and ZnT1 expression in HCASMC

When we examined the effects of hypoxia/reoxygenation on NRF2 targeted NQO1 and glutamate cysteine ligase modifier subunit (GCLM) expression in HCASMC pre-adapted for 5d to 18 or 5 kPa O_2_, NQO1 protein expression trended to be lower in cells adapted to 5 kPa O_2_ and was significantly lower in cells exposed to hypoxia (12 h) and reoxygenation (12 h) under 5 kPa O_2_ compared to 18 kPa O_2_ (Supplementary Fig. 4A). In contrast, negligible changes in GCLM (Supplementary Fig. 4B) and zinc transporter 1 (Zn efflux transporter [48]), (Supplementary Fig. 4C) expression were detected in cells pre-adapted to 18 or 5 kPa O_2_ and exposed to hypoxia/reoxygenation.

### 3.6 Effects of Zn chelation or supplementation on ROS generation in HCASMC under hypoxia/reoxygenation

**Fig. 7:**
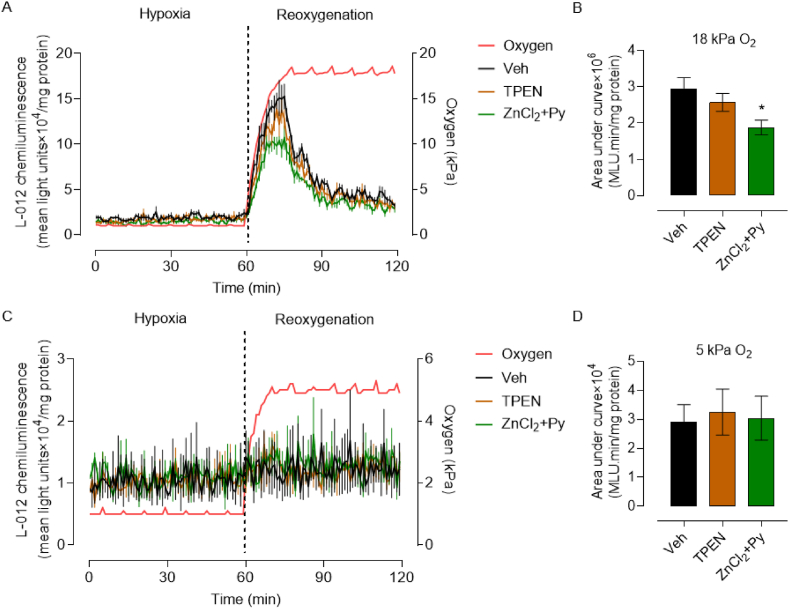
Zn supplementation attenuates reoxygenation-induced reactive oxygen species generation in HCASMC under 18 kPa O_2_ **A and C**, Representative L-012 luminescence traces in HCASMC pre-adapted for 5d to 18 or 5 kPa O_2_. Cells were treated for 16 h with Veh (0.01% DMSO), TPEN (1.25 μM) or ZnCl_2_ (10 μM) + pyrithione (Py, 0.5 μM). Cells were then incubated with L-012 and transferred to an O_2_-regulated plate reader. L-012 luminescence was detected in the plate reader gassed with 18 or 5 kPa O_2_, with O_2_ then reduced to 1 kPa and reoxygenation under either 18 or 5 kPa O_2_, respectively. The red line indicates the pericellular O_2_ levels within the plate reader. **B and D**, Area under curve summary of reoxygenation-induced L-012 luminescence changes. L-012 signal in panel B (18 kPa O_2_) denotes area under the curve x10^6^ and in panel D (5 kPa O_2_) area under the curve x10^4^. Data denote mean ± S.E.M., n = 4 independent cultures, one-way ANOVA followed by Bonferroni's multiple comparisons test, *P < 0.05. (For interpretation of the references to color in this figure legend, the reader is referred to the Web version of this article.)

As we established that Zn activates NRF2 signaling in HCASMC under 18 and 5 kPa O_2_, we next investigated whether Zn chelation or supplementation attenuates reoxygenation-induced free radical generation. HCASMC were pre-adapted for 5d to 18 or 5 kPa O_2_ and then pre-treated for 16 h with TPEN (1.25 μM) or ZnCl_2_ (10 μM) + pyrithione (0.5 μM). After incubation with L-012, cells were exposed to hypoxia (1 h) and reoxygenation under 18 or 5 kPa O_2_ respectively in an O_2_-controlled plate reader. Under low medium Zn concentration (2 μM), TPEN had negligible effects on reoxygenation stimulated L-012 luminescence, whereas Zn supplementation attenuated reoxygenation-induced reactive oxygen species generation in cells under 18 kPa O_2_ (Fig. 7A and B). As in our previous study in brain microvascular endothelial cells [34], negligible changes in L-012 luminescence were detected after reoxygenation of HCASMC under 5kPaO_2_ (Fig. 7C and D).

## 4 Discussion

The present study, to our knowledge, is the first to investigate the effects of Zn chelation or supplementation on total intracellular Zn content, NRF2 regulated antioxidant gene transcription and hypoxia/reoxygenation induced ROS generation in HCASMC cultured long-term under standard hyperoxia (18 kPa O_2_) or physiological normoxia (5 kPa O_2_). Zn supplementation significantly increased total intracellular Zn content in HCASMC under 18 but not 5 kPa O_2_, whilst metallothionein-1 mRNA expression was upregulated in cells under either pericellular O_2_ level. Although Zn supplementation increased NRF2 nuclear accumulation in cells under 18 and 5 kPa O_2_, NRF2 targeted HO-1 mRNA and protein expression induced by Zn supplementation was attenuated in HCASMC under 5 kPa O_2_, with a similar trend observed for NQO1. Notably, hypoxia/reoxygenation induced free radical generation was inhibited by Zn supplementation in HCASMC only under 18 kPa O_2_, consistent with our observation of enhanced ROS generation and antioxidant enzyme expression in cells under hyperoxia.

In view of contractile and synthetic phenotypes in vascular smooth muscle cells, our study is the first to investigate the effects of pericellular O_2_ levels on the phenotype of HCASMC, highlighting a more contractile phenotype in cells under 5 compared to 18 kPa O_2_. Badran et al. recently reviewed the role of ROS as modulators of vascular smooth muscle phenotype and suggested that excessive ROS generation induces a synthetic phenotype associated with disease, whilst physiological ROS levels are associated with a contractile phenotype [49]. Moreover, activation of NRF2 in response to 7-ketocholestrol stimulated ROS generation has been shown to maintain mouse coronary arterial smooth muscle cells in a differentiated state [50]. Our finding of increased calponin-1 expression in HCASMC under 5 kPa O_2_ thus further underpins the importance of physiologically relevant O_2_ levels for cell culture models *in vitro.*

Using ICP-MS, we report novel evidence that Zn supplementation (10 μM, 16 h) induced increases in total Zn content in HCASMC are markedly attenuated in cells under physiological normoxia (5 kPa) compared to hyperoxia (18 kPa O_2_). The Zn ionophore pyrithione enhances Zn uptake in oligodendrocyte progenitor cells [45], cardiac H9c2 cells [44,51] and isolated papillary muscle [52]. In the present study, HCASMC were treated with concentrations of ZnCl_2_ + pyrithione and TPEN that had negligible effects on cell viability (see Supplementary Fig. 1). The Zn chelator TPEN (1.25 μM) had negligible effects on total intracellular Zn content. It is possible that the low basal Zn concentration in the media for HCASMC may in part explain the lack of a statistical difference between vehicle and TPEN treated cells. Total Zn content may not be altered following treatment of HCASMC with TPEN, since ICP-MS analysis would also detect Zn chelated by TPEN. As reported in many other studies [53], TPEN would be expected to decrease bioavailable Zn levels.

Labile Zn (free Zn^2+^) levels can increase in cells within seconds, minutes or hours after exogenous Zn supplementation with transcriptional regulation of Zn transporters observed over a longer time scale [54,55]. Zn ‘muffling’ reactions modulate transient changes in Zn^2+^ after Zn supplementation via zinc transporters (e.g. ZIP1-14 importers and ZnT1-10 exporters) and zinc-binding proteins to maintain a tight control of intracellular Zn^2+^ levels [[55], [56]]. ZIP2 and ZIP12 have been implicated in endothelial and smooth muscle responses to vascular Zn deficiency [35] and, although ZIP12 expression is low in normal vascular tissue, exposure of human pulmonary vascular smooth muscle cells to hypoxia upregulates ZIP12 mRNA whilst ZIP6, ZIP7 and ZIP10 are unaffected [57]. In rat aortic smooth muscle cells, Zn supplementation (25–50 μM) has been shown to decrease ZnT3 and ZnT10 expression and increase senescence [58]. Based on limited information available on the effects of Zn supplementation on ZIP and ZnT transporters in different vascular smooth muscle cell types, further characterization Zn transporters in cells maintained under physiological normoxia and exposed to hypoxia/reoxygenation is warranted.

Oxidative stress and hypoxia can induce release of Zn from metallothioneins (MT), leading to activation of MTF-1 and increased expression of MTs and ZnT1 [27,59]. In the present study, MT1 mRNA expression was significantly upregulated by Zn supplementation in HCASMC under 18 and 5 kPa O_2_, consistent with previous studies in human pulmonary vascular smooth muscle cells conducted under ambient air [35]. We previously reported that MT1 and ZnT1 mRNA/protein expression in HCASMC was unaffected by changes in pericellular O_2_ levels, and in the present study establish that ZnT1 protein expression is affected negligibly by Zn supplementation in HCASMC under 18 or 5 kPa O_2_ (see Supplementary Fig. 4C).

NRF2 plays a key role in maintaining cellular redox homeostasis [[5], [6], [7], [8]], and previous studies have established that Zn influences NRF2 activation in different cell types cultured in standard incubators under hyperoxia (18 kPa O_2_), including endothelial cells [60], human renal tubule cells [25,61], retinal pigment epithelial cells [26], IMR-32 neuroblastoma cells [62], murine spinal cord neurons [63] and human peripheral blood mononuclear cells [64]. NRF2 protein levels are maintained relatively low under physiological conditions due to rapid ubiquitination and proteasomal degradation mediated by Keap1 [5,6] and GSK3β phosphorylation of NRF2, leading to β transducin repeats-containing proteins (βTrCP)-mediated degradation and Fyn-mediated nuclear exclusion [7,65]. Recent evidence in human peripheral blood mononuclear cells suggests that Zn supplementation inhibits HDAC3 activity, using a cell-free assay, and decreases Keap1 mRNA expression without altering NRF2 protein levels [64]. By contrast, Zn supplementation (3d) of human leukemia monocytic THP-1 cells has negligible effects on NRF2 and Keap1 mRNA expression [66].

Our findings establish that upregulation of HO-1 in response to Zn supplementation is attenuated in HCASMC adapted to physiological normoxia (5 kPa O_2_) (see Fig. 5), consistent with negligible changes in total intracellular Zn content under 5 kPa O_2_ (Fig. 3B). Kaufman et al. implicated Zn-mediated degradation of Bach1, the mammalian repressor of HO-1, in the regulation of HO-1 expression by Zn in neuroblastoma cells [62]. In this context, specific loss of Bach1 in murine vascular smooth muscle inhibits neointimal hyperplasia and remodelling following vascular injury [67]. The interaction of intracellular Zn^2+^ with His-225, Cys-226 and Cys-613 within Keap1 is associated a conformational change in Keap1 leading to inhibition of NRF2 ubiquitination [23,24]. Keap1, acting as a Zn sensor and Zn-binding protein, can also release Zn from Cys-273 and Cys-288 under oxidative stress [22,24]. Zn supplementation has been shown to phosphorylate Akt and/or ERK1/2 and inhibit GSK3β in human renal tubule cells and mouse kidney [25], neonatal ventricular myocytes [17] and H9c2 cardiac cells [51], which would enhance NRF2 nuclear accumulation and downstream antioxidant gene expression [6,7,68]. Notably, ZIP6-induced Zn influx in MCF-7 cells has been associated with inhibition of GSK3β [69].

We previously reported that culturing vascular and other cell types under different O_2_ levels significantly alters NRF2 regulated redox signaling [19,31,34,70,71], noting that long-term adaptation of human and murine endothelial cells to physiological normoxia (5 kPa O_2_) attenuates NRF2 transcriptional activation of HO-1 and NQO1 [19,31,34]. Our present findings demonstrate that Zn supplementation enhances NRF2 nuclear accumulation in HCASMC under 18 and 5 kPa O_2_, noting that Zn induced upregulation of HO-1/NQO1 protein expression is attenuated in cells under 5 kPa O_2_. Under these experimental conditions, Zn supplementation only protected HCASMC against reoxygenation induced free radical generation during culture under 18 kPa O_2_ (see Fig. 6, Fig. 7), confirming the protection afforded by the NRF2 inducer sulforaphane against reoxygenation induced O_2_^−.^ generation in brain microvascular endothelial cells [34] and highlighting the lower redox stress experienced by cells under physiological normoxia [29,30]. In this context, Kelmanson et al. reported only a slight decrease in H_2_O_2_ levels in the cytosol and mitochondria of neurons exposed to hypoxia for 30min using genetically encoded Hyper7 biosensors [72]. Notably, pretreatment of rat neonatal cardiomyocytes with Zn/pyrithione attenuates hypoxia/reoxygenation induced superoxide generation [12] and dietary Zn/pyrithione supplements affords protection against coronary ischemia/reperfusion injury [11].

Our finding that Zn supplementation activates NRF2 signaling in HCASMC and can attenuate hypoxia/reoxygenation induced ROS generation provides a basis for design and screening of therapeutic drugs for treatment of coronary heart disease. Given the caveats concerning cell culture under hyperoxia [29,30,[73], [74], [75], [76], [77]], the potential influence of pericellular O_2_ on chemical fluorescence probes [78] and our present findings that effects of Zn supplementation and the phenotype of HCASMC are altered by physiological normoxia, we encourage researchers to study interactions between Zn and redox signaling in cells under physiological O_2_ levels. Moreover, such experiments should also consider zinc concentrations in culture media and the effect that pericellular O_2_ has on cellular zinc metabolism and signaling involving or being affected by zinc ions.

## Authors contributions

F.Y., M.J.S. and G.E.M. conceptualized the study; F.Y., M.J.S., A.G., A.M. developed the methodology; F.Y. performed and analyzed all experiments and A.G. conducted the ICP-MS analyses; F.Y. and G.E.M. wrote the manuscript, which all authors reviewed. G.E.M. is the guarantor of this study, with responsibility for the integrity of the data and data analysis.

## Declaration of competing interest

The authors declare that they have no known competing interests that could have influenced the study.

## Data Availability

No data was used for the research described in the article.
