# Supplementary material for: Vascular protection afforded by zinc supplementation in human coronary artery smooth muscle cells mediated by NRF2 signaling under hypoxia/reoxygenation
Source: Redox Biol. 2023 Jun 7;64:102777. doi: 10.1016/j.redox.2023.102777 (PMC10363453; doi:10.1016/j.redox.2023.102777)
Supplement: Multimedia component 1 [file mmc1.docx]

**Redox Biology … (2023) 102777 – Supplementary Data**

**Vascular protection afforded by zinc supplementation in human coronary artery smooth muscle cells mediated by NRF2 signaling under hypoxia/reoxygenation**

Fan Yang^1*^, Matthew J. Smith^1^, Alexander Griffiths^2^, Alexander Morrell^2^, Sarah J. Chapple^1^, Richard C.M. Siow^1^, Theodora Stewart^3^, Wolfgang Maret^4^ and Giovanni E. Mann^1*^

*^1^ King’s British Heart Foundation Centre of Research Excellence, School of Cardiovascular and Metabolic*

*Medicine & Sciences, Faculty of Life Sciences & Medicine, King’s College London, 150 Stamford Street,*

*London SE1 9NH, U.K.*

*^2^ London Metallomics Facility, Faculty of Life Sciences & Medicine, King’s College London, U.K.*

*^3^ Research Management & Innovation Directorate (RMID), King’s College London. U.K.*

*^4^ Departments of Biochemistry and Nutritional Sciences, School of Life Course & Population Sciences, Faculty*

*of Life Sciences & Medicine, King's College London. U.K.*

**Supplementary Table 1 - qPCR primers**

| **Gene name** | **Forward (5’→3’)** | **Reverse (3’→5’)** | **Amplicon(bp)** |
| --- | --- | --- | --- |
| RPLP0 | CAGATTGGCTACCCAACTGTT | GGGAAGGTGTAATCCGTCTCC | 98 |
| TBP | CACGAACCACGGCACTGATT | TTTTCTTGCTGCCAGTCTGGAC | 89 |
| SDHA | CTACAAGGTGCGGATTGATG | ATAGGACAGGGTGTGCTTCC | 100 |
| HO1 | GGCTTCAAGCTGGTGATGG | AGTAGACAGGGGCGAAGACTG | 100 |
| NQO1 | GCACTGATCGTACTGGCTCA | GGTCCTTCAGTTTACCTGTGATG | 172 |

*Abbreviations:* RPLP0, ribosomal protein lateral stalk subunit P0; TBP, TATA-binding protein; SDHA, succinate dehydrogenase complex, subunit A; HO-1, heme oxygenase-1, NQO1, NAD(P)H quinone dehydrogenase 1


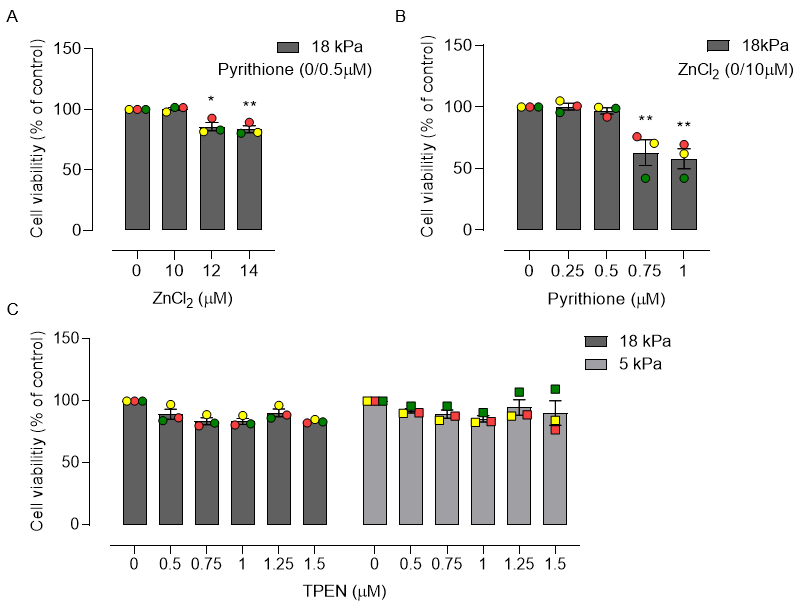


Supplementary Fig.1 Viability of HCASMC following treatment with ZnCl_2_/pyrithione or TPEN

HCASMC pre-adapted for 5d to 18kPa O_2_ were seeded in triplicate at a concentration of 10^4^ cells/well in 96-well plates and cultured for 48h. Cells were then treated for 16h in the absence (control medium) or presence of different concentrations of ZnCl_2_/pyrithione or TPEN and an MTT assay used to assess cell viability. **A**, Pyrithione (0.5µM) + ZnCl_2_ (10, 12 or 14µM). **B**, ZnCl_2_ (10µM) + pyrithione (0.25, 0.5, 0.75 or 1µM). **C**, TPEN (0.5, 0.75, 1.0, 1.25 or 1.5µM). Data denote mean ± S.E.M., n=3-4 independent cultures (color-coded), one-way or two-way ANOVA with Tukey’s or Bonferroni’s multiple comparisons test, **P*<0.05, ***P*<0.01. *Abbreviations*: TPEN, N,N,N′,N′-tetrakis (2-pyridylmethyl) ethylenediamine


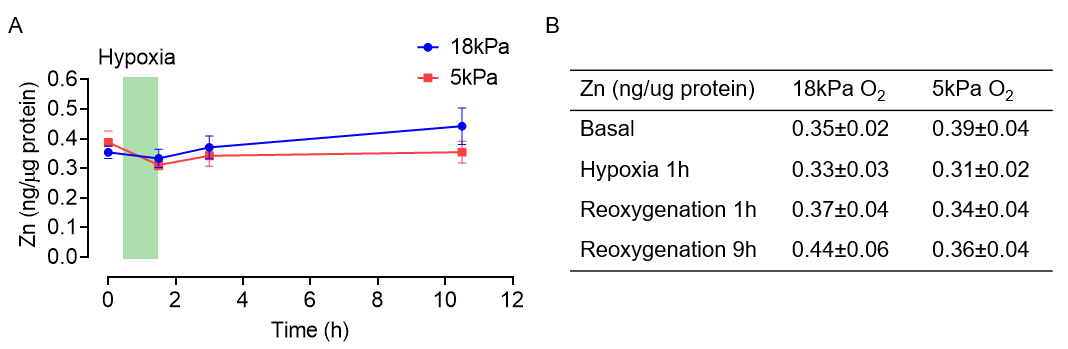


Supplementary Fig. 2 Effects of hypoxia/reoxygenation on total intracellular Zn content in HCASMC under 18 or 5kPa O_2_

HCASMC were pre-adapted for 5d to 18 or 5kPa O_2_ and cells exposed to hypoxia and reoxygenation. **A**, ICP-MS analysis of total intracellular Zn content in HCASMC under basal conditions, hypoxia (1kPa, 1h) and reoxygenation (for 1h and 9h) under 18 or 5kPa O_2_, respectively. Changes between 18kPa or 5kPa and 1kPa O_2_ required 30 min with cells maintained in the O_2_-controlled workstation. **B**, Summary of total Zn content for each condition and time point. Data denote mean ± S.E.M., n=4-10 independent cultures, two-way ANOVA with Tukey’s multiple comparisons test.

Supplementary Fig. 3 Effects of hypoxia/reoxygenation on intracellular glutathione levels in HCASMC under 18 or 5kPa O_2_

HCASMC were pre-adapted for 5d to 18 or 5kPa O_2_ and intracellular glutathione (GSH) then measured under basal conditions (control), hypoxia (1h) only or followed by reoxygenation (HR) for 1h or 24h under 18 or 5kPa O_2_, respectively. Data denote mean ± S.E.M., n=4 independent cultures (color-coded), two-way ANOVA with Tukey’s or Bonferroni’s multiple comparisons test, **P*<0.05.


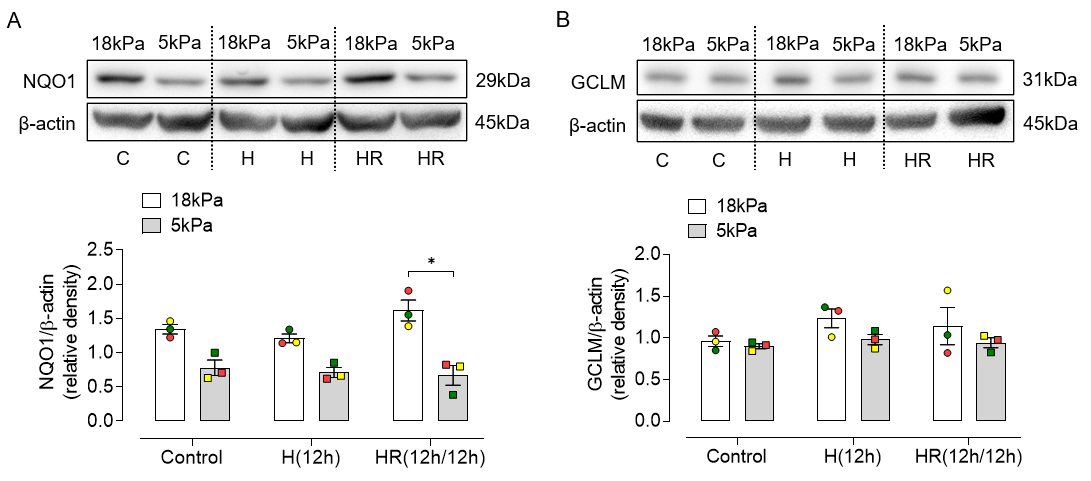


C


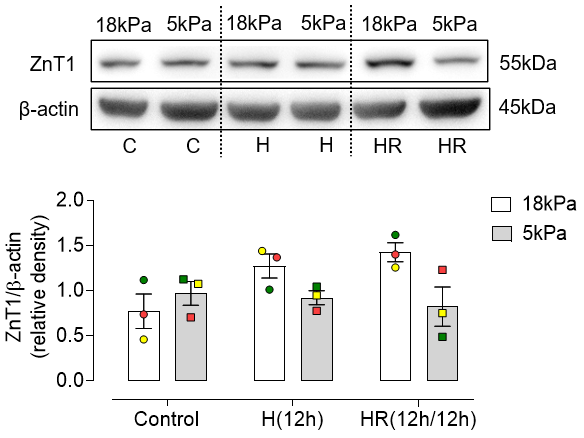


Supplementary Fig. 4 NQO1, GCLM and ZnT1 expression in HCASMC under hypoxia-reoxygenation

HCASMC were pre-adapted for 5d to 18 or 5kPa O_2_ (control) and then subjected to hypoxia (H) for 12h or hypoxia for 12 h followed by reoxygenation for 12h (HR) under 18 or 5kPa O_2_, respectively. Representative immunoblots and densitometric analyses of NQO1 (**A**), GCLM (**B**) and ZnT1 (**C**) expression relative to β-actin. Data denote mean ± S.E.M., n=3 independent paired cultures (color-coded), two-way ANOVA with Tukey’s multiple comparisons test, **P*<0.05.
